# NRF2: A crucial regulator for mitochondrial metabolic shift and prostate cancer progression

**DOI:** 10.3389/fphys.2022.989793

**Published:** 2022-09-23

**Authors:** Brigitta Buttari, Marzia Arese, Rebecca E. Oberley-Deegan, Luciano Saso, Arpita Chatterjee

**Affiliations:** ^1^ Department of Cardiovascular and Endocrine-metabolic Diseases and Aging, Istituto Superiore di Sanità, Rome, Italy; ^2^ Department of Biochemical Sciences “A. Rossi Fanelli”, Sapienza University of Rome, Rome, Italy; ^3^ Department of Biochemistry and Molecular Biology, University of Nebraska Medical Center, Omaha, NE, United States; ^4^ Department of Physiology and Pharmacology ‘‘Vittorio Erspamer”, Sapienza University of Rome, Rome, Italy

**Keywords:** prostate cancer, metabolism, Nrf2, therapy resistance, oxidative stress, cancer progression, mitochondria

## Abstract

Metabolic alterations are a common survival mechanism for prostate cancer progression and therapy resistance. Oxidative stress in the cellular and tumor microenvironment dictates metabolic switching in the cancer cells to adopt, prosper and escape therapeutic stress. Therefore, regulation of oxidative stress in tumor cells and in the tumor-microenvironment may enhance the action of conventional anticancer therapies. NRF2 is the master regulator for oxidative stress management. However, the overall oxidative stress varies with PCa clinical stage, metabolic state and therapy used for the cancer. In agreement, the blanket use of NRF2 inducers or inhibitors along with anticancer therapies cause adverse effects in some preclinical cancer models. In this review, we have summarized the levels of oxidative stress, metabolic preferences and NRF2 activity in the different stages of prostate cancer. We also propose condition specific ways to use NRF2 inducers or inhibitors along with conventional prostate cancer therapies. The significance of this review is not only to provide a detailed understanding of the mechanism of action of NRF2 to regulate oxidative stress-mediated metabolic switching by prostate cancer cells to escape the radiation, chemo, or hormonal therapies, and to grow aggressively, but also to provide a potential therapeutic method to control aggressive prostate cancer growth by stage specific proper use of NRF2 regulators.

## 1 Introduction and purpose of the review

Nuclear factor erythroid 2-related factor 2 (NRF2) protein is encoded by the human NFE2L2 gene and is a member of basic leucine zipper transcription factor protein families (He et al., 2020). In an oxidatively stressed cellular environment, NRF2 avoids Keap1-mediated degradation, translocates to the nucleus, and binds to antioxidant response elements (ARE) in the promoter regions of genes, which produce cellular antioxidants and detoxifying proteins (He et al., 2020). Other than this canonical job, NRF2 also maintains mitochondrial health to control oxidative stress (Dinkova-Kostova and Abramov, 2015). As mitochondrial health and function is the prime regulator for cellular metabolism, NRF2 is also a crucial controller for metabolic health. Therefore, NRF2 can be considered a master regulator for cellular defense against oxidative stress and resulting metabolic changes.

Overall redox homeostasis helps to maintain physiological metabolism and other cellular functions. In pathological conditions, such as cancers, the metabolic rate increases to sustain the higher energy demands of rapidly proliferating cancer cells (Han et al., 2020). Higher metabolic activity in the cancer cells produces increased amounts of reactive oxygen species (ROS) from the mitochondrial electron transport chain. At the same time, due to overactivity and metabolic exhaustion, mitochondria become damaged, which produce more ROS in the cellular environment (Dakubo et al., 2006). Therefore, redox balance is disrupted in the cancer cells, which then adapt to survive in a high oxidative environment and stabilize NRF2 as one of the major weapons to combat ROS. Therefore, increased NRF2 activity is crucial for cancer cells to prosper in a highly oxidizing environment.

The purpose of this review is to summarize the functions by which NRF2 regulates mitochondrial health and metabolism in varying redox environments, and therapy responses in prostate cancer (PCa) and its microenvironment. PCa has been chosen as the model of this discussion because initially PCa is sensitive to therapy but in the advanced stages, PCa becomes therapy resistant, which can result in metastatic growth. The early and late forms of PCa also have different ROS environments and metabolic preferences, which reflect the different mitochondrial activity at different stages of PCa (Cutruzzola et al., 2017). Therefore, PCa will give us the opportunity to evaluate NRF2-mediated regulation of mitochondrial health and metabolism in different stages of the disease. This discussion may help to illuminate the proper use of NRF2 related therapies in PCa treatment in the future.

In this review, we will discuss the status of ROS in PCa progression, metabolic alteration, and therapy resistance. Then we will discuss on the canonical function of NRF2 in cellular defense and in mitochondrial health maintenance. Finally, we will discuss the role of NRF2 in the mitochondria-mediated alterations in PCa progression and therapy resistance.

## 2 Mitochondrial function: A critical regulator for prostate cancer severity

### 2.1 PCa progression, ROS, and mitochondria

Increased oxidative stress is required for increased invasiveness in metastatic PCa (B. Kumar et al., 2008). Prostate cancer cells can produce ROS from NADPH oxidases (NOX), mitochondrial metabolism as well as endoplasmic reticulum stress (Khandrika et al., 2009; Costanzo-Garvey et al., 2022). The following studies support that oxidative stress increases as PCa progresses and that the increased oxidative stress support PCa aggressiveness. In one study, reduction of ROS by diphenyleneiodonium chloride blocked PCa cell proliferation by blocking ERK, p38 and AKT signaling and caused cyclin B-dependent G2M cell cycle arrest (B. Kumar et al., 2008). In another study, increased oxidation of macromolecules such as, lipids, proteins and nucleic acid were used to measure oxidative stress and showed increased oxidation correlates with aggressiveness of PCa (Zha et al., 2005; Kalinina et al., 2022). Polymorphisms in the genes related to oxidative stress and antioxidant response were also investigated in the PCa patient’s blood and polymorphisms that change the function of NQO1 and SOD2 are positively associated with prostate carcinogenesis (Gong Z et al., 2021; Z. Zhang et al., 2019). SOD, GSH, and CAT activity were significantly lower and serum levels of malondialdehyde were significantly higher in prostate cancer patients as compared to healthy controls (Ahmed Amar et al., 2019). In another study, SOD activity was found to be higher in PCa patients as compared to healthy population whereas levels of thiobarbituric acid reactive substances (TBARS), protein carbonylation, were significantly higher in PCa patients (Battisti et al., 2011). This indicates that SOD protein was enhanced to try to combat the high levels of oxidative stress in tumors, but the antioxidant scavenging system was still overwhelmed. ROS mediated oxidation and post translational modifications in the macromolecules cause a defective downstream signaling to increase oncogenic signaling in the advanced PCa (Shiota et al., 2012; T. Wu et al., 2020; Yin et al., 2021).

Normal prostate epithelial cells depend on glycolysis (Cutruzzola et al., 2017) and have low ROS levels (Khandrika et al., 2009). After transformation of normal prostate cells to the initial PCa stage, cells begin to depend on mitochondria for metabolism (Mamouni et al., 2021). As the energy demand increases due to higher proliferation of PCa cells, mitochondrial exhaustion generates higher amounts of ROS in localized PCa. At the metastatic PCa state, energy demand further increases and the PCa cells produce more ROS from the extensive metabolic processes (Han et al., 2020). Reactivation of glycolysis, ongoing TCA cycle and initiation of fatty acid metabolism are the sources of energy for highly proliferating metastatic PCa cells (Tousignant et al., 2019). PCa cells display a neuroendocrine phenotype at this later stage. Therefore, functional alterations in mitochondria are crucial for PCa progression. Mitochondrial DNA mutations including a 3.4 kb mitochondrial genome deletion was reported as a biomarker for benign to malignant to metastatic progression of PCa (Maki et al., 2008; Xiao et al., 2018). As PCa progresses, mitochondrial activity and ROS levels increase.

### 2.2 Metabolic shift in PCa progression

Unlike other cancers, PCa aggressiveness is not linearly dependent on glycolysis. Aggressive PCa cells also depend on mitochondrial oxidative phosphorylation (OXPHOS) (C. L. Chen et al., 2021). Normal prostate epithelium accumulates zinc, which inhibits mitochondrial aconitase activity. Aconitase helps to consume citrate for the tricarboxylic acid (TCA) cycle. Therefore, in normal prostate epithelial cells, the TCA cycle is truncated, and the unused citrate is released to the extracellular space. Excess extracellular citrate and intracellular zinc levels result in the dependency of normal prostate epithelial cells on glycolysis. As PCa progresses from prostatic intraepithelial neoplasia to metastasis, there are decreased zinc levels (Ho and Song, 2009; Costello and Franklin, 2011), which enhances mitochondrial aconitase activity and the total levels of secreted citrate becomes low as it is utilized by the TCA cycle (Uo et al., 2020). In the more advanced PCa state, along with OXPHOS, citrate also is converted into acetyl-CoA to fuel fatty acid synthesis (Uo et al., 2020). Moreover, in advanced PCa, stromal fibroblasts cause a reverse Warburg effect in PCa cells and promote PCa cell growth (Ippolito J et al., 2016). Therefore, in PCa progression, OXPHOS metabolism, fatty acid metabolism, zinc and citrate shuttling are crucial factors.

### 2.3 Therapy resistance of PCa and mitochondria

Transformation of PCa progresses from normal prostate to benign prostate hyperplasia (BPH), to prostatic intraepithelial neoplasia (PIN), to localized PCa, to metastatic castration resistance PCa (CRPC), and neuroendocrine (NEPC) type PCa. Depending on the clinical stage, therapy options for localized PCa are active surveillance, prostatectomy, local radiotherapy, androgen deprivation therapy (ADT) and a combination of two or more of these therapies. After metastasizing to local or distant organs, the therapy options for these aggressive PCa cells are ADT and chemotherapies. Often failure of these therapies results in radioresistant, chemoresistant, CRPC and NEPC, which causes PCa related deaths. Therapies, such as, radiotherapy, kill PCa cells via ROS but the cells escape therapy-mediated cell death and become adapted to in high ROS environment, which results in relapse of the disease. Therefore, increased oxidative environment is a crucial factor for therapy resistant growth.

#### 2.3.1 ADT resistance in PCa

In the early stages of PCa, the cancer is androgen sensitive. As the disease progresses, PCa becomes androgen independent, accumulates more ROS, and at the final advanced stages of PCa, reactivation of non-canonical androgen receptor (AR) signaling in CRPC, and chemical ADT resistance enhances glycolysis (Moon et al., 2011; Butler et al., 2016). Therefore, in addition to OXPHOS and fatty acid metabolism, ADT resistant PCa also depends on glycolysis. Metabolic reprograming in PCa is tightly linked to androgen signaling in PCa. Reactivation of androgen signaling in CRPC also leads to increased citrate production by increasing the substrate pools for citrate synthase, acetyl-CoA and oxaloacetic acid (Uo et al., 2020). Advanced PCa also uses lipids for their energy production. PCa that is resistant to androgen receptor signaling inhibitors, such as, enzalutamide or abiraterone, are dependent on ceramide and sphingosine kinase signaling (H. M. Lin et al., 2021). Therefore, metabolic switching and alteration of ROS levels are vital factors for ADT resistant PCa.

#### 2.3.2 Radioresistance in PCa

As in the case of ADT, the late stage PCa become resistant to radiotherapy as well. The degree of radioresistance depends on the internal oxidative state. Radiation causes oxidative stress in cancer cells as well as in the normal cells surrounding the tumor. Radioresistant PCa often causes aggressive transformation to NEPC and metastasis, finally resulting in death of the PCa patients. NEPC growth is dependent on mitochondrial metabolism-mediated acidic extracellular pH (Ippolito L et al., 2016). Radiation damages mitochondria in PCa cells, and metabolic alterations after radiation are obvious in radioresistant PCa. As discussed above, maintaining mitochondrial health is necessary for therapy resistant growth of PCa. A major antioxidant and protector of overall mitochondrial health is MnSOD. Suppression of MnSOD sensitizes PCa cells to radiation (Holley et al., 2010). Mitochondrial metabolic switching in radioresistant PCa needs to be studied more extensively.

#### 2.3.3 Chemoresistance in PCa

Mitochondrial metabolic rewiring in chemoresistance and chemotherapy induced side effects are well reported (Mamouni et al., 2021). Cisplatin, an anticancer agent used for PCa treatment, is a DNA damaging agent, which also damages mitochondrial DNA, resulting in reduced mitochondrial function (Marrache et al., 2014; Cocetta et al., 2019). Cisplatin-induced side effects cause a reduction in the quality of life in PCa patients, which can be mitigated by reducing oxidative stress (Aminuddin et al., 2020). Docetaxel is a chemotherapeutic agent against cell cycle progression for advanced PCa. Docetaxel resistant PCa cells are more efficient in utilizing glucose, glutamine, and lactate by OXPHOS as compared to docetaxel sensitive cells (Ippolito L et al., 2016). In docetaxel resistant cells, the use of OXPHOS blockers, such as metformin, inhibit proliferation and invasiveness by pushing the cells towards glycolysis (Ippolito L et al., 2016). Mitochondrial lipid metabolism, especially cholesterol metabolism, is upregulated in CRPC and in enzalutamide resistant PCa (S. Kim and Kim, 2021). Therefore, in advanced chemoresistant PCa, mitochondrial OXPHOS, lipid metabolic pathways and altered ROS levels are crucial targets for therapeutic intervention.

### 2.4 Cancer stem cell growth, ROS and PCa progression

A major reason for therapy resistant PCa growth is cancer stem cell (CSC) proliferation, de-differentiation, and plasticity. Therefore, specific targeting of CSC would lead to enhancement of treatment efficacy and reduce the chances of tumor recurrence. CSCs rely on OXPHOS as a main source of energy production; therefore, this OXPHOS dependency can be used to selectively target CSCs in combination with PCa therapies. Treatments of PCa by OXPHOS complex I inhibitors, force PCa cells to compensate the energy need by glycolytic switch. This may reduce CSC activity during treatment resistance and increase CSC sensitivity to conventional therapies (Mayer et al., 2015). Stem cell fate and function are regulated, in part, by the cellular redox state and metabolic activities. Stem cells need low levels of ROS, which are critical for regulation of stem cell quiescence and self-renewal. Therefore, to be active in a high ROS environment and supply proliferative cells after therapy, CSCs need to be protected from high oxidative stress. Thus, mitochondrial metabolism and redox status have recently received attention for advanced stage PCa therapy, especially for targeting CSC (Sotgia et al., 2018).

In this section, we have discussed the importance and interrelationship of increased oxidative stress and mitochondrial metabolic pathways, especially preferential usage of glycolysis, OXPHOS and lipid metabolism with PCa progression and therapy resistance. From this knowledge we speculate that PCa cells and other cells in a PCa tumor microenvironment, use a potent survival mechanism to cope and prosper in this increasing oxidative stress environment as PCa progresses. NRF2 is a potential candidate as it is a master regulator of cellular defense against oxidative stress and metabolism. In the next sections, we will discuss the role of NRF2 in regulating oxidative stress, mitochondrial metabolic shifting in PCa progression and therapy resistance.

## 3 NRF2: The master regulator of cellular defense

### 3.1 Domain structure of NRF2

**FIGURE 1:**
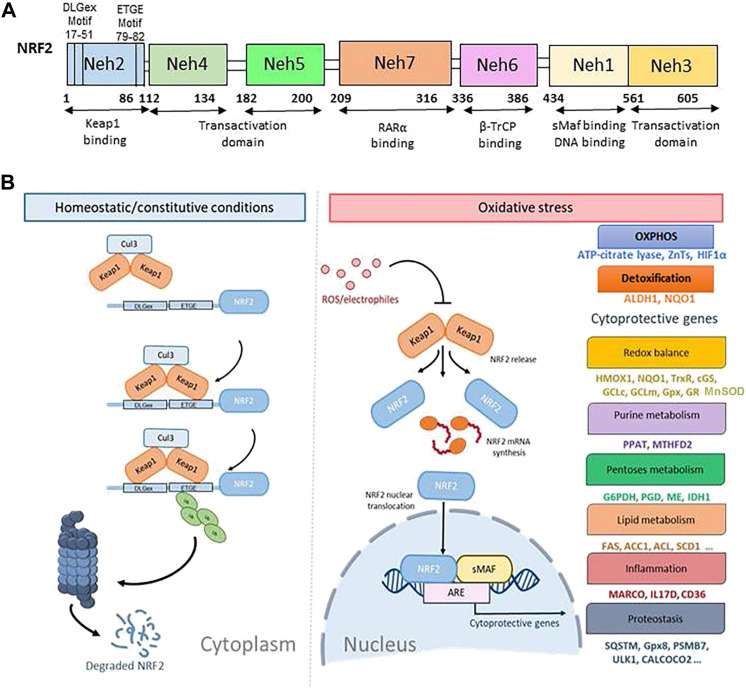
The canonical regulatory pathways of NRF2 signaling. **(A)**. Domain structure of NRF2 (NF-E2-related factor 2), member of the Cap’n’collar (CNC) transcription factor family, consists of 605 amino acids organized in seven highly conserved functional domains, known as Neh1-Neh7. **(B)**. Under homeostatic/constitutive conditions, a continuous degradation of NRF2 protein in the cytoplasm is achieved, through the formation of a complex of involving E3 ubiquitin ligase containing the regulatory cysteine rich Keap1 protein. Binding of the Keap1 homodimer is mediated by the two NRF2 sequences, ETGE and DLGex, located in the Neh2 domain, which leads to its ubiquitylation and proteasomal degradation. When Keap1 is challenged with ROS or electrophiles, critical cysteines are modified, a non-functional KEAP1 complex is generated, and NRF2 degradation is blocked. Thus, *de-novo* synthesized NRF2 is stabilized and imported to the nucleus where it activates the ARE-mediated transcription of cytoprotective genes.

NRF2 is a master regulator of cellular defense against oxidative and xenobiotic stress. NRF2, a member of the Cap’n’collar (CNC) transcription factor family and contains seven Neh (NRF2-ECH homology) domains, namely Neh1-Neh7 (Figure 1A). The Neh1 domain includes a basic region-leucine zipper (bZIP) structure, which regulates DNA binding by favoring NRF2 dimerization with small Maf proteins (sMafs) (Katsuoka and Yamamoto, 2016) and a nuclear localization signal (NLS) that regulates the nuclear import of NRF2 (Theodore et al., 2008). Neh2 and Neh6 contain degradation signals, which allow NRF2 targeting to proteasomal degradation by Kelch-like ECH-associated protein1 (KEAP1) and β-transducin repeats-containing proteins (β-TrCP) (Rada et al., 2011). The N-terminal regulatory domain, Neh2, contains seven lysine residues and two peptide-binding motifs (DLG and ETGE) that influence binding with different proteins responsible for NRF2 ubiquitination and its proteasomal degradation under normal physiological conditions (M. Lin et al., 2015). The Neh3, Neh4, and Neh5 are transactivation domains, which regulate the binding of NRF2 with other coactivators (Katoh et al., 2001; Nioi et al., 2005). The Neh5 domain controls the cytoplasmic localization of NRF2 (Krajka-Kuzniak et al., 2017). The Neh6 domain, with serine-rich residues, acts as a negative regulatory domain and binds with β-TrCP for NRF2 ubiquitination (Rada et al., 2012). The Neh7 domain binds to the retinoic X receptor α (RXRα), a repressor of NRF2; thus, contributing to the inhibition of NRF2-ARE signaling pathway (H. Wang et al., 2013).

### 3.2 Physiologic regulation of NRF2 and the KEAP1/NRF2/ARE pathway

The expression of NRF2 is tightly regulated. Under normal homeostatic conditions, NRF2 mRNA is constitutively expressed (Stewart et al., 2003) and is sequestered within cytoplasm by its regulator KEAP1 (Figure 1B). KEAP1 is a cysteine-rich protein, which is divided into five domains, an N-terminal region (NTR), a Tramtrack-Bric-a-Brac (BTB) domain, a central intervening region (IVR) with a nuclear export signal (NES) regulating the cytoplasmic localization of KEAP1, six Kelch repeats, and a C-terminal domain (CTR) (Ogura et al., 2010). In the canonical regulatory pathways of NRF2 signaling, NRF2 is constitutively ubiquitinated by KEAP1, an adaptor component of the Cullin 3 (Cul3)-based ubiquitin E3 ligase complex, which leads to its ubiquitylation and proteasomal degradation. The high rate of NRF2 ubiquitination and degradation in non-stressed cells are largely cell-type dependent due to varying concentrations of the KEAP1 protein (Itoh et al., 1999). Recently, p97, an ATP-dependent segregase, was identified as a canonical negative regulator of NRF2 needed for its efficient proteasomal degradation (Tao et al., 2017). In the noncanonical pathway, under pathological oxidative stress conditions, the clearance of damaged organelles, long-lived proteins, or misfolded proteins by autophagy is compromised (Eskelinen and Saftig, 2009). The autophagy chaperone p62/Sequestosome-1 accumulates and competitively binds to Keap1, leading to increased NRF2 signaling (Jain et al., 2010; Komatsu et al., 2010; Lau et al., 2010). KEAP1 is a cysteine rich protein and senses oxidative stress via the cysteine residues (such as, Cys151, Cys273, and Cys288). Oxidation of cysteines causes conformational changes in KEAP1, which results in release and stabilization of NRF2 to maintain ROS homeostasis (Saito et al., 2016).

In stress-inducing conditions (Kaspar et al., 2009; Stefanson and Bakovic, 2014; Shaw and Chattopadhyay, 2020), cells activate the NRF2/KEAP1/ARE pathway (Figure 1B). Intracellular ROS or electrophiles readily react with cysteine-thiols in KEAP1 and stabilize NRF2/KEAP1 complex conformation thus blocking NRF2 ubiquitylation. In this way, free KEAP1 is not regenerated and *de-novo* synthesized NRF2 is imported to the nucleus wherein it heterodimerizes with sMaf and binds to a regulatory enhancer sequence ARE; thus, promoting the expression of antioxidant and detoxifying genes and down-modulating the production of pro-inflammatory mediators (Saha et al., 2020; D. D. Zhang and Hannink, 2003). ARE sequences (5′-RTGACnnnGC-3′) are present in the promoters of different genes such as glutamate-cysteine ligase catalytic (GCLC) and modifier (GCLM) subunit, NAD(P)H quinone oxidoreductase 1 (NQO1), heme-oxygenase-1 (HMOX1), sulfiredoxin1 (SRXN1), glutathione S-transferase (GST), multidrug resistance-associated proteins (MRPs), and UDP-glucuronosyltransferase (UGT) (A. Kumar and Mittal, 2017). The presence of ARE in the HMOX1 promoter allows for the NRF2 mediated synthesis of the corresponding protein, heme oxygenase (HO-1), the enzyme that catalyzes the first and rate limiting step in heme degradation (Choi and Alam, 1996).

Two models were reported in the literature to explain the regulation of NRF2 stability. The first, also known as the ‘‘hinge and latch’’ model, postulates that KEAP1 interaction with the ETGE domain of NRF2 acts as a hinge, while a weaker interaction with the DLGex motif of the Neh2 acts as a latch (Kansanen et al., 2012). According to this model, the disruption of the latch abolishes NRF2 ubiquitination (Taguchi et al., 2011; Kansanen et al., 2013). The two-site binding mode is supported by the observation that somatic mutations in various cancer cells occur with very high-frequency in the DLGex and ETGE motifs of NRF2 (Shibata et al., 2008; Cancer Genome Atlas Research, 2012). These mutations alter the two-site binding of KEAP1-NRF2 and lead to constitutive accumulation of NRF2, supporting cancer cell growth (Taguchi and Yamamoto, 2017). By using competitive inhibition NMR experiment, Horie et al. (2021) demonstrated that the hinge-latch mechanism is actively utilized in the NRF2 activation by pharmacological KEAP1-NRF2 protein-protein interaction inhibitors (PRL295 and NG262), but not by electrophilic NRF2 inducers. The later observation suggests that the existence of other mechanisms of NRF2 activation, in addition to the hinge-latch. The same authors also examined the binding regions involved in the interaction of KEAP1 with p62. They found phosphorylated p62/Sequestosome-1 peptide interferes with binding of DLGex of Neh2 to KEAP1; thus, opening the latch site, which suppresses efficient ubiquitination and rapid degradation of NRF2 (Horie et al., 2021). The second model, also known as KEAP1-independent regulation, postulates that in normal conditions, Neh6 domain binds to the DSGIS and DSAPGS motifs of the β-TrCP, which in turn is a substrate receptor for the Skp1-Cul1-Rbx1/Roc1 ubiquitin ligase complex that drives NRF2 ubiquitination (Chowdhry et al., 2013). The NRF2 phosphorylation in the Neh6 domain by glycogen synthase kinase-3 regulates the recognition by β-TrCP (Rada et al., 2011). Both discussed models suggest that newly synthesized NRF2 translocates into the nucleus (Taguchi et al., 2011; Kansanen et al., 2013).

### 3.3 Intranuclear regulation of NRF2

The abundance of NRF2 inside the nucleus is tightly controlled. After NRF2-induced defensive genes, the Src kinase family member, Fyn, phosphorylates NRF2 at tyrosine residue 568, which leads to a chromosomal region maintenance-1 (Crm-1) mediated nuclear export and degradation (Courtneidge et al., 1993). Alternatively, NRF2 is negatively regulated by glycogen synthase kinase-3β (GSK-3β), a kinase that sensitizes cells for cell death. The phosphorylation of NRF2 by GSK-3β promotes nuclear exclusion of NRF2; therefore, preventing binding and activation of the AREs (Salazar et al., 2006).

The BTB and CNC homology 1 (BACH1) transcription factor is a negative NRF2 competitor and molecular sensor of intracellular heme, it competes with NRF2 for its binding sites on DNA (Dhakshinamoorthy et al., 2005). BACH1 is a MAF-related transcriptional repressor, it forms heterodimers with small Maf (MafK and MafG) proteins on DNA leading to the repression of ARE-mediated gene expression and induction (Igarashi et al., 1998) that is conserved and ubiquitously expressed in tissues (Oyake et al., 1996; Sun et al., 2002). In the presence of an antioxidant, BACH1 becomes phosphorylated at Tyr 486 and is exported from the nucleus and degraded in the cytosol; thus, allowing NRF2 access to the ARE (Kaspar and Jaiswal, 2010). The antioxidant also leads to an increase in synthesis of BACH1 that is imported into the nucleus to achieve the normal level of BACH1 and repression of antioxidant gene expression. In oxidative stress conditions, heme is released from hemoproteins, leading to more oxidative stress. As a sensor of heme, BACH1 binds heme and it induces not only its nuclear export but also its polyubiquitination and degradation (Zenke-Kawasaki et al., 2007), thereby losing its activity as a repressor.

NRF2 plays a key role in combating pathologic ROS formation as well as inflammatory and metabolic responses by coordinating the activity of various proteins; it induces the expression of antioxidants as well as cytoprotective genes through the regulation of multiple signaling pathways. In the next section we will discuss the role of NRF2 in mitochondrial metabolism and maintenance of overall health of the cell in increased oxidative stress in initial and advanced stages of PCa.

## 4 NRF2: A mitochondrial protector

Properly orchestrated reduction of mitochondrial ROS, maintenance of ATP production and a balance in fission, fusion and mitophagy result in healthy mitochondrial function. NRF2 activation increases PINK/Parkin mediated mitophagy and eliminates damaged mitochondria from cells (Gumeni et al., 2021). Mitochondrial ROS and NRF2 have an inverse relationship (Kasai et al., 2020; Tsushima et al., 2020; Hartwick Bjorkman and Oliveira Pereira, 2021). Induction of NRF2 decreases mitochondrial ROS and, thus, cancer cells use NRF2 to grow in a high ROS environment. With the help of PGC1α, NRF2 increases mitochondrial biogenesis to supply healthier mitochondria (Gureev et al., 2019). NRF2 maintains mitochondrial membrane potential, OXPHOS, ATP synthesis, fatty acid synthesis and oxidation (Dinkova-Kostova and Abramov, 2015). It is reported that NRF2 plays a very crucial role in overall ATP production and utilization (Holmstrom et al., 2013). NRF2 activation is directly related to increased mitochondrial membrane potential, ATP levels, and enhanced efficiency of OXPHOS (Wang Y et al., 2018). Advanced staged PCa cells commonly take advantage of NRF2’s ability to protect mitochondrial health. For instance, the initial stages of PCa cells generally have low NRF2 levels and may take advantage of this strategy for energy production without increasing mitochondrial ROS. NRF2 deficient cells increase ATP production through glycolysis, which is then used by the F1F0-ATPase for maintenance of the mitochondrial membrane potential (Holmstrom et al., 2013). In addition, the levels and activities of the OXPHOS complexes were unaffected in NRF2 deficient cells (Holmstrom et al., 2013). Therefore, in NRF2 deficient cells, a basal level of mitochondrial respiration also takes place without large increases in mitochondrial ROS levels. NRF2 is one of the main regulators of mitochondrial function. However, mitochondrial metabolism was not completely silenced in NRF2 deficient cells. Therefore, it is possible that some of the NRF2 downstream genes, such as, NQO-1 and HO-1, can be expressed though NRF2 independent pathways (Sun et al., 2002; Korashy and El-Kadi, 2008; Kang et al., 2014), and can still regulate mitochondrial metabolism in NRF2 deficient cells. The NAD^+^/NADPH ratio, mitochondrial ROS levels and ATP production can be regulated by NQO-1 (Haefeli et al., 2011; J. Kim et al., 2013). HO-1 can regulate mitochondrial oxygen consumption rate, electron flow *via* electron transport chain, mitochondrial metabolic substrate utilization (Carr et al., 2020). AP1 has overlap in function with NRF2 as it binds to the same ARE sequence to upregulate the antioxidant system. Therefore, the role of AP1 in the NRF2 deficient cells needs to be evaluated to determine redox balance. Since NRF2 increases as PCa progresses, for a mitochondrial metabolism dependent cancer, such as, PCa, NRF2 can be considered as a vital regulator for metabolic changes and progression of PCa.

## 5 NRF2: A double-edged sword for prostate cancer

As we have previously discussed, oxidative stress increases during PCa progression and therapy resistance, which provides a cue to the aggressive PCa cells to activate more NRF2 to adapt to higher oxidative stress. Since mitochondrial metabolic shift and NRF2 activity are tightly connected with PCa aggressiveness, then blanket use of an NRF2 activator or inhibitor is not a good choice for all PCa patients. For example, testosterone analogs and specific competitive inhibitors of AR, such as, finasteride and durasteride, are used to treat BPH. Finasteride and durasteride were used in two large clinical trials for PCa (PCPT and REDUCE) and showed significant reduction of initial PCa incidence (Thompson et al., 2003; Andriole et al., 2010). However, unexpectedly, at later times, occurrence of high grade PCa (Gleason scores 7–10) were significantly increased among the subjects given finasteride or durasteride as compared to the placebo group (Azzouni and Mohler, 2012). Further studies showed the basal expression of NRF2 was higher in androgen-resistant PCa cells (DU-145 and PC3) as compared to androgen sensitive PCa cells (LNCaP), and finasteride treatment selectively increased NRF2 expression in DU-145 and PC-3 cells, but not in LNCaP cells. This study proposed that finasteride-mediated induction of NRF2 in high grade PCa cells is at least partly responsible for the increased risk for high grade PCa after finasteride treatment (Yun et al., 2013). Therefore, expression and activity of NRF2 have a stage dependent effect on PCa growth.

### 5.1 NRF2 in PCa progression and metastasis

Disruption of NRF2 activity, elevated ROS and increased DNA damage have been reported as responsible factors for the oncogenic transformation at the initial stage of human PCa (Frohlich et al., 2008). In the initial stages, PCa cells do not depend on NRF2 for survival as the ROS levels in the initial stage PCa are low. Increased ROS from initial therapy, such as, radiotherapy or ADT, cause initial cell death as the tumor does not possess an adequate antioxidant system to handle the increased ROS levels. But the cells that survive the initial therapy will grow in a highly oxidative niche due to enhanced ROS from PCa cell metabolism and radiotherapy and become dependent on NRF2-mediated antioxidant system to survive. These cells will eventually become therapy resistant and become more aggressive. In support of this, high glucose treatment increased ROS levels and caused cell death in the androgen sensitive PCa cells, LNCaP, which is representative of early stage of PCa. Expression levels of NRF2 and its target genes were significantly lower in high glucose treated LNCaP cells as compared to the cells in normoglycemia (J. Y. Chen et al., 2019). However, in aggressive PC3 cells, basal NRF2 expression and activity was higher (Bellezza et al., 2017). Organo-selenium based NRF2 activator compounds decreased oxidative stress in LNCaP cells and inhibited cell growth via cell cycle arrest (Terazawa et al., 2010). Corosolic acid, an epigenetic inducer of NRF2 transcription, reduced anchorage-independent growth of wild type TRAMPC PCa cells, an aggressive model of advanced PCa, but not NRF2 knockout TRAMPC cells (J. Yang et al., 2018). The absence of NRF2 increases the oxidative stress and therapy resistance in the cells.

### 5.2 Mutations and polymorphisms that regulate NRF2 function in PCa

NRF2 levels depend on the activity of Keap1 and p62 as discussed in *NRF2: The master regulator of cellular defense*. Therefore, along with direct NRF2 mutations, mutations in the NRF2 regulatory genes, Keap1 and p62, also affect NRF2 function. Mutation in speckle-type POZ protein (SPOP) is common in PCa, SPOP binds to p62 and releases it from Keap1. Keap1 then becomes available for NRF2 binding, which reduces NRF2 activity. In PCa, mutated SPOP cannot bind to p62, which makes Keap1 engaged with p62 instead of NRF2, which results in oncogenic stabilization of NRF2 in PCa (Shi et al., 2022). The presence and binding of p62 with Keap1 leads to decreased degradation of NRF2 in PCa and results in apoptosis resistance, invasion, and proliferation (Jiang et al., 2020). Promoter hypermethylation and aberrant splicing of Keap1 in PCa cells increase levels of NRF2 and PCa cell growth (P. Zhang et al., 2010). Stage specific NRF2 expression and activity in PCa can also be explained in a study, where promoter methylation of NRF2 were studied in different stages of PCa. This study showed NRF2 promoter methylation was highest in the initial stage of the disease, such as, BPH and LNCaP cells as compared to the more aggressive stages, such as, recurrent ADT-resistant tumors (Khor et al., 2014). Therefore, along with direct mutation and promoter methylation of NRF2, mutations in Keap1 and p62, which can increase stabilization of NRF2, are crucial regulatory factors for PCa progression and therapy resistance. We may propose to analyze these genes for mutations along with the conventional preventive examinations for PCa in the future.

### 5.3 NRF2 in metabolic changes in PCa

**FIGURE 2:**
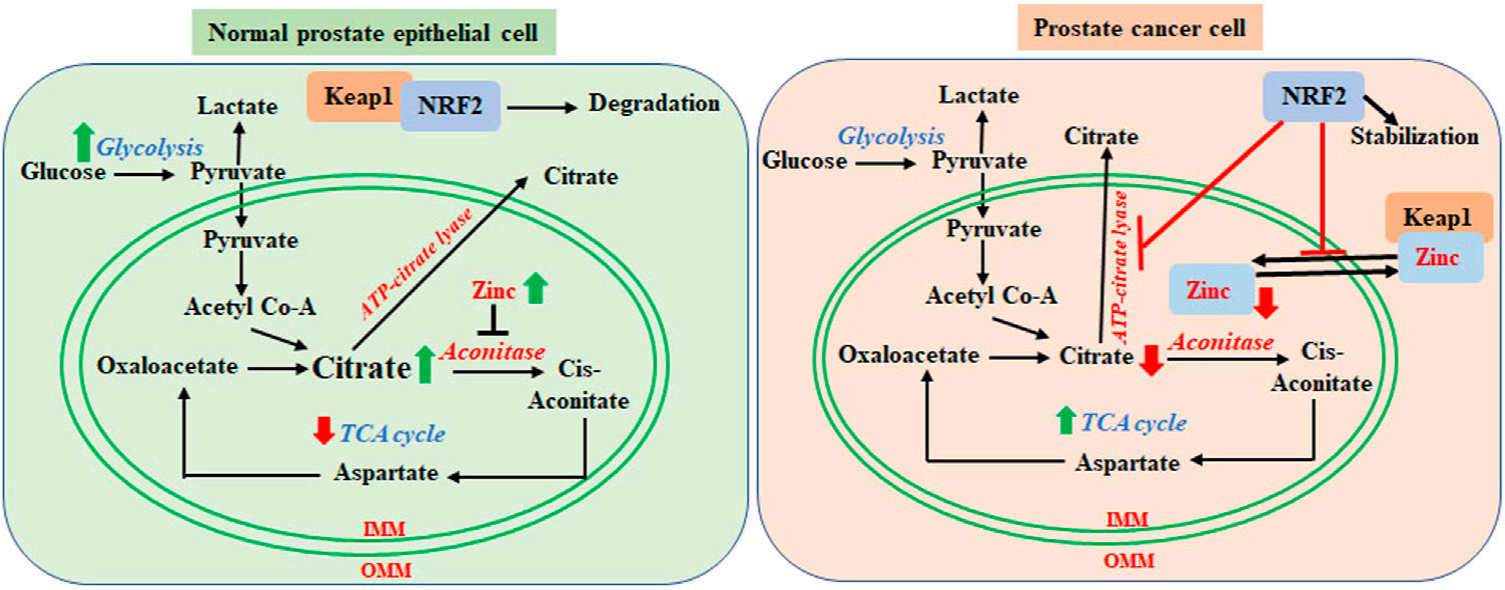
Regulatory role of NRF2 in metabolic switching from normal prostate cell to prostate cancer cells. In normal glycolytic prostate epithelial cells, mitochondria contain higher levels of zinc, which inhibits aconitase activity. This results in the accumulation of citrate in the mitochondria and export outside of mitochondria, which decrease TCA cycle-mediated energy generation. In the cytosol, Keap1 sequesters NRF2 for degradation. In the prostate cancer cells, zinc levels are lower in the mitochondria. Zinc translocates to the cytosol and competitively binds to Keap1, which releases NRF2. Stabilized NRF2 inhibits mitochondrial import of zinc and ATP-citrate lyase mediated citrate lysis and export. Lower zinc levels enables mitochondrial aconitase to utilize citrate via the TCA cycle. Mitochondrial retention of citrate enables fatty acid synthesis by acetyl CoA. PCa cells hereby rely on the TCA cycle and fatty acids for energy generation in the more advanced stages.

As we discussed before, energy demand, ROS and NRF2 dependency increase with PCa advancement, and the PCa cells begin utilizing all available sources for energy production from glycolysis to OXPHOS to lipid metabolic pathways to survive. Metabolic rewiring and high NRF2 levels are interlinked factors that promote the growth of advanced PCa. It was previously reported that in *in vitro* and *in vivo* models of leukemia those induce OXPHOS also enhance NRF2 expression and activity (Khan et al., 2018). NRF2 can reactivate OXPHOS in OXPHOS-reliant tumor cells (Zdralevic et al., 2018). NRF2 is also positively associated with PGC1α in various cells, which is a major regulator of fatty acid synthesis. Therefore, in advanced PCa, increased NRF2 activity may be positively associated with increased OXPHOS and fatty acid synthesis. However, there are few studies published about the role of NRF2 on specific OXPHOS enzymes and subcellular and extracellular shuttling of citrate in PCa. We have previously discussed zinc and citrate-mediated switching between glycolysis and TCA during PCa progression. NRF2 activation increases expression of zinc transporters (Ishida and Takechi, 2016), which efflux zinc from the intracellular organelles (such as mitochondria) and inhibits its reuptake. Therefore, higher NRF2 levels in advanced PCa help to keep zinc levels low inside the cells and in the mitochondria (Ishida and Takechi, 2016), which enhances the TCA cycle. On the other hand, zinc supplementation increases nuclear translocation, transcriptional activity of NRF2 and antioxidant gene expression (Y. Chen et al., 2015; Cortese et al., 2008; Ha et al., 2006; B. Li et al., 2014; Mehta et al., 2011). In a stressed cell, Keap1 senses zinc and undergoes a conformational change after zinc binding, which releases NRF2 from Keap1 and stabilizes it (Dinkova-Kostova et al., 2005; McMahon et al., 2010; McMahon et al., 2018). Therefore, NRF2 activation and cellular zinc levels are linked together. We may surmise that NRF2 mediated zinc transportation and efflux may have a key role in the metabolic switching of PCa progression via citrate release. Along with the above-mentioned zinc mediated metabolic switching, NRF2 is also crucial for citrate shuttling, metabolic alteration, and PCa progression. ATP-citrate lyase, which can be inhibited by NRF2 (Yates et al., 2009; Kitteringham et al., 2010; Dinkova-Kostova and Abramov, 2015), cleaves citrate to produce acetyl-CoA and activates fatty acid synthase. Fatty acids relocate into the mitochondria via CD36 and Carnitine O-Palmitoyltransferase (CPT1) to serve as the substrate for fatty acid oxidation in early stages of PCa progression. Therefore, NRF2 retains citrate inside the mitochondria by inhibiting ATP-citrate lyase (Yates et al., 2009; Kitteringham et al., 2010; Dinkova-Kostova and Abramov, 2015). We have summarized the role of NRF2 in metabolic shifting of PCa cells in Figure 2.

NRF2 is a crucial regulator for other major metabolic pathways. A glycogen metabolizing enzyme, glycogen phosphorylase, brain form (PYGB) is a growth promoting enzyme in PC3 cells. Suppression of PYGB reduced NRF2 signaling and increased ROS, which resulted in apoptosis (Wang Z et al., 2018). The major rate limiting enzymes of the pentose phosphate pathway, G6PD and 6PGD, are target genes of NRF2 (Hagiwara et al., 2021). Higher expression and activity of NRF2 in high grade PCa cells is also useful for the cell to efficiently metabolize a pro-apoptotic molecule, 4-hydroxynonenal, to a nontoxic metabolite and escape apoptosis (Pettazzoni et al., 2011). During the shift from initial therapy (radiation and androgen deprivation) sensitive growth to therapy resistant aggressive growth, PCa cells also shift their metabolism. Increased PGC1α levels and decreased HIF1α are the major regulators for this metabolic shift towards OXPHOS and lipid synthesis. The interrelationship between NRF2 and PGC1α in mitochondrial biogenesis and metabolic health maintenance is well studied. AMPK phosphorylates both PGC1α and NRF2 (Gureev et al., 2019). Phosphorylated PGC1α directly promotes mitochondrial biogenesis (Jager et al., 2007; Canto et al., 2009), whereas phosphorylated NRF2 translocates to the nucleus and maintains mitochondrial health by expressing antioxidant enzymes. PGC1α promotes NRF2 nuclear localization by inhibiting GSK3β, whereas NRF2 directly induces PGC1α expression by binding to its promoter (Gureev et al., 2019). Sulforaphane, a NRF2 inducer, enhances mitochondrial biogenesis and fragmentation in PCa cells (Negrette-Guzman et al., 2017). Hypoxia promotes metastasis and therapy resistance in the tumors. HIF1α is an important metabolic regulator during hypoxia, which can be regulated by NRF2. NRF2 binds to an enhancer element upstream of the HIF1α gene and promotes HIF1α gene expression (Jin et al., 2020). Decreasing NRF2 in a hypoxic tumor reduces hypoxia-mediated deleterious metabolic activity of HIF1α (Toth and Warfel, 2017; Lee et al., 2019; Jin et al., 2020). Therefore, an increase in NRF2-mediated mitochondrial biogenesis is a key event for the metabolic shift and increase in aggressiveness of PCa cells.

From these reports, we suggest that inhibition of NRF2 in the advanced PCa, will increase zinc levels in the cancer cells and export citrate from the mitochondria, which will reduce TCA mediated metabolism and fatty acid synthesis. The absence of NRF2 will also compromise mitochondrial health *via* reduction of PGC1α and HIF1α activity. As a result, mitochondrial metabolism-dependent aggressive, therapy-resistant PCa growth may be controlled. However, glutathione oxidation is a crucial metabolic regulator of the pentose phosphate pathway (Amara et al., 2020). Levels of NAD and NADPH regulate overall oxidative stress and metabolic shifts in PCa. Although NRF2 regulates NAD/NADPH ratio via NQO1 and total decrease in oxidative stress, NOX4 also can regulate the NAD/NADPH ratio independent of NRF2 (Papadimitriou et al., 2015; Mejia et al., 2018; Qiu et al., 2022). Therefore, glutathione oxidation and NOX4 activity needs to be considered during investigation on PCa metabolic shift, especially in the reduced NRF2 conditions, which have not carefully studied in the literature.

### 5.4 NRF2 in PCa therapy resistance

Enhanced NRF2 activity in cancer cells can accumulate oncogenic metabolites and detoxify or shuttle the anticancer therapies out of the tumor cells (van der Merwe et al., 2021). Therefore, in modulating ROS levels and metabolic alteration in PCa therapy resistance, NRF2 is a vital target.

#### 5.4.1 ADT resistance and NRF2

NRF2 levels are low in early AR sensitive PCa cells but increase in advanced PCa cells (Schultz et al., 2014). In AR-sensitive LNCaP cells, a model of early stage PCa, NRF2 levels were low and overexpression of NRF2 in LNCaP cells significantly reduced AR activity basally and after DHT stimulation. However, in more aggressive PCa cells, such as, C4-2B, NRF2 overexpression reduced AR transactivation only after dihydrotestosterone (DHT) stimulation (Schultz et al., 2014). It has been reported in a non-cancer model that AR physically interacts with NRF2, and this interaction was increased by DHT (Hu et al., 2022). PCa cells expressing full-length androgen receptor (AR-FL) are ADT-sensitive and cells expressing ligand-independent, constitutively active AR-V7 variant are ADT resistant. Enzalutamide, an AR antagonist, is a potent anti-PCa drug used to reduce AR mediated PCa growth. Aggressive enzalutamide resistant PCa growth is a common clinical challenge. Activation of NRF2 increased enzalutamide efficacy in enzalutamide-resistant PCa cells by degrading both the AR-FL and AR-V7 variants (Khurana et al., 2017). NRF2 reduces AR signaling by transcriptional suppression of AR gene expression (S. H. Kim and Singh, 2009), protein degradation (Gibbs et al., 2009) or epigenetic regulation (W. Li et al., 2018; Shi et al., 2009). At the early stages of PCa, NRF2 levels are low, AR activity is high, and cells display AR sensitive growth. In this stage, if NRF2 levels are enhanced in combination with ADT, increased efficacy of ADT will be observed. However, in the later stages, when cells are habituated to high endogenous NRF2 levels and low AR dependent growth, the use of an NRF2 activator may not be advantageous. If the cells depend on non-canonical signaling mediated by AR, NRF2 may help to reduce AR levels and make the cells more sensitive to ADT. However, if the cells are not dependent on AR, NRF2 activation will not be helpful when combined with ADT, rather, NRF2 activation will likely promote cancer cell survival by lowering ROS-mediated cellular damage. Bardoxolone-methyl, which enhances NRF2 activity and decreases AR-FL in LNCaP, C4-2B and 22Rv1 cells and decreases AR-V7 expression in 22Rv1 cells, enhances enzalutamide efficacy (Khurana et al., 2020). After ADT, human prostatic glands and carcinoma tissues displayed higher NRF2 activity, increased senescence, and increased expression of proinflammatory cytokines (Kawata et al., 2017). Therefore, ROS and NRF2 levels, along with AR activity, should be considered important regulatory factors for use of ADT for PCa.

#### 5.4.2 Radiosensitization and NRF2

As the intracellular NRF2 levels depend on the need to cope with high oxidative stress, radioresistant PCa cells also have higher levels of NRF2. Radiotherapy-mediated increased ROS enhance NRF2 levels in PCa as well as tumor associated normal cells. Reduction of NRF2 levels can sensitize cells to radiotherapy (Jayakumar et al., 2014). Differential NRF2 regulation after radiation by phytochemicals, such as, parthenolide, in normal vs. cancer cells is reported. Parthenolide selectively oxidizes Keap1 in the normal prostate epithelial cells, while reducing Keap1 in PCa cells. This leads to the upregulation of NRF2 mediated pro-survival pathway in normal cells and suppression of the NRF2 pathway in cancer cells after radiation (Xu et al., 2013). An interesting finding in the TRAMPC PCa model showed that ADT lowers basal ROS levels in PCa cells via upregulation of NRF2, which sensitizes the tumor to radiotherapy (Liu et al., 2015). Radiation damages mitochondria, and radioresistant PCa cells protect mitochondrial damage after radiation by enhancing MnSOD, a well-known NRF2 regulated enzyme. Thus, inhibiting NRF2 would decrease MnSOD levels leading to the sensitization of PCa to radiotherapy. Another NRF2 regulated mitochondrial protein, aldehyde dehydrogenase 1A1 (ALDH1A1), is involved in aldehyde detoxification and elicits radioresistance in PCa through AKT activation (Wakamiya et al., 2022). Suppression of NRF2 leads to reduction of aldehyde dehydrogenase 1 and radioresistance (Duong et al., 2017). In support of these studies, inhibition of NRF2 in DU-145 cells enhanced radiation killing (P. Zhang et al., 2010). These data indicate that after radiotherapy, radioresistant cells are NRF2 dependent, and suppression of NRF2 in these cells may lead to cell death.

#### 5.4.3 Chemosensitization and NRF2

As was previously discussed, the high oxidative environment in advanced PCa also results in chemoresistance. Enhanced NRF2 activity in a high ROS environment promotes chemoresistance by controlling oxidative stress in the cells after chemotherapy. Patients with CRPC often show resistance to the chemotherapeutic agent, cabazitaxel, which is commonly used for treatment of metastatic PCa. NRF2 transcription was enhanced in cabazitaxel resistant PCa cells but not in the cabazitaxel sensitive cells. Inhibition of NRF2 in cabazitaxel resistant cells and overexpression of NRF2 in cabazitaxel sensitive cells reversed cabazitaxel response phenotypes (Endo et al., 2021). Therefore, in advanced chemoresistant, NRF2 dependent cells, NRF2 inhibitors may help to reduce chemoresistance. However, the NRF2 inducer, sulforaphane, increases the efficacy of cisplatin and doxorubicin by mitigating their toxicity without affecting their anticancer effects (Calcabrini et al., 2020). Therefore, if the PCa cells are not dependent on NRF2 for growth, a NRF2 inducer may also increase chemotherapy efficacy. Mitochondrial lipid metabolism is a crucial factor for chemoresistance (Cao, 2019). NRF2 activity alters overall lipid oxidative stress in PCa cells (Pettazzoni et al., 2011). Hence, NRF2 could be a crucial modulator for chemoresistance in PCa via modulating mitochondrial lipid metabolism also.

### 5.5 NRF2 in PCa stem cell growth

After cancer therapy, CSC need a lower ROS environment to maintain the stem-like cell phenotypes. NRF2 is a potential factor for PCa stem cell maintenance and activity after therapies. Higher levels of NRF2 help to maintain low levels of ROS and stemness properties. NRF2 induces spheroid formation, migration, epithelial to mesenchymal transition and other CSC phenotypes. Therefore, high NRF2 levels are crucial for stem cell survival, self-renewal, and plasticity (Dai et al., 2020). Chronic exposure of inorganic arsenic as an environmental toxin activates NRF2, which increases PCa incidence via increased self-renewal and decreased differentiation of human prostate stem-progenitor cells (Xie et al., 2020). MUC1-C, an oncogenic protein, enhances lineage plasticity in PCa progression. Mechanistically, MUC1-C binds with NRF2 and forms a complex, which maintains the redox balance, pluripotency factor expression, and lineage plasticity of PCa stem like cells in CRPC and NEPC (Hagiwara et al., 2021). Aldehyde dehydrogenase (ALDH1A1) is a CSC biomarker and higher expression of ALDH1A1 is associated with drug resistance and tumor growth (D. Kim et al., 2018). NRF2 expression and activity were increased in CSC, which also express high levels of ALDH1A1 (D. Kim et al., 2018). Silencing of NRF2 reduced expression of ALDH1A1 and ALDH3A1, which reduced chemoresistance and increased the efficacy of the anticancer drugs (Duong et al., 2017; Matsumoto et al., 2021). Survival of drug resistant high aldehyde dehydrogenase expressing tumor cells was significantly reduced by NRF2-silencing (D. Kim et al., 2018). Therefore, NRF2 is a vital factor in maintaining PCa stem cell function and reduction of NRF2 in PCa stem like cells may increase therapy efficacy.

### 5.6 NRF2 in PCa tumor microenvironment maintenance

Normal cells surrounding the tumor, such as, fibroblasts and immune cells, oppose cancer initiation and tumor growth through healthy physiological defense mechanisms. Cells in the tumor microenvironment maintain their normal redox state via NRF2, which leads to antitumor functions. Therefore, increases in NRF2 in the normal cells in the tumor microenvironment help to maintain the antitumor environment and inhibit cancer growth at the very initial phase of cancer growth. However, in the case of aggressive tumor growth, cancer cells impair the normal function of cells in the microenvironment in favor to promote their own survival. The normal antitumor activity of fibroblasts and the immune cells are converted to pro-tumor phenotypes through differentiation into cancer associated fibroblasts and tumor promoting immune cells. We have reported that manganese porphyrins protect normal prostate cells from radiation-induced fibrosis by enhancing NRF2 activity, while inhibiting PCa growth after radiation (Tong et al., 2016; Chatterjee et al., 2017; Shrishrimal et al., 2017; Chatterjee et al., 2018; Chatterjee et al., 2020; Shrishrimal et al., 2020; Zhu et al., 2020). Increased levels of ROS are quite common in the aggressive tumor environment caused by higher metabolism and by the cancer cell itself. Higher NRF2 activity in the tumor associated fibroblasts and pro-tumor immune cells will promote survival in a higher ROS environment. Pro-inflammatory cytokines stimulate mesenchymal stem cells (MSC) to increase expression of angiogenic factors via NRF2 activation and enhance PCa growth (K. Q. Yang et al., 2017). NRF2 increases tumor growth via heightened angiogenic and hypoxic response through the production of HIF1α and VEGFA (Funes et al., 2014) after transforming MSCs to CSCs. It was also reported that NRF2 expression significantly varies between Gleason score 3 and 4 in the tumor microenvironment of PCa (Georgescu et al., 2016). Therefore, higher NRF2 activity in the tumor microenvironment, which is primarily a defense mechanism of the body against cancer, can be converted to a tumor promoting tool in the aggressive tumor microenvironment. It is also reported that the NRF2 activator, sulforaphane, acts pro-oxidatively in human primary T cells, which inhibits T cell activation and effector functions (Liang et al., 2019). Therefore, non-targeted NRF2 activation with immunotherapy may not be an efficacious therapeutic option.

## 6 NRF2 modulators in pre-clinical PCa research

The goal is to reduce NRF2 activity in the cancer cells, but not in normal cells. When PCa progresses to advanced stages, it adapts to survive in high ROS environment with the help of NRF2. Downregulation of NRF2 activity reduces the viability of advanced staged PCa cells, DU145 and PC3. Downregulating NRF2 increases ROS, oxidation of nucleic acids and lipids, decreases the activity of antioxidant enzymes, and increases the unfolded protein response and endoplasmic reticulum stress in advanced stage PCa (J. Yu et al., 2021). A plant-based polyphenol fraction upregulated ROS levels by decreasing NRF2 and increasing monoamine oxidase A activity, both in androgen-dependent LNCaP and androgen-independent PC3 and DU145 cells but not in normal cells. This polyphenol fraction also reduced PC3-tumor xenograft in NOD-SCID mice alone and in synergy with paclitaxel (Ghosh et al., 2021). Cisplatin based PCa therapy results in testicular injury, which is caused by enhanced ROS and inflammation. Roflumilast enhances NRF2 activity and protects the normal testicular cells but not the PC3 cells from cisplatin-based toxicity (Abdel-Wahab et al., 2021). Therefore, a NRF2 modulator can improve the overall efficacy of existing PCa therapy. We have also shown that manganese porphyrins increase NRF2 expression only in the normal prostate fibroblasts and reduces fibrosis after radiation, while reducing PCa growth after radiation (Tong et al., 2016; Chatterjee et al., 2018; Zhu et al., 2020). To cope with the oxidatively stressed environment, PCa cells also use alternative NRF2 pathway activators, such as long noncoding RNA, TUG1, which enhances expression of NRF2 target genes. Inhibition of TUG1 reduced proliferation, migration, and invasion of PCa cells (G. Yang et al., 2020). In the presence of endoplasmic reticulum (ER) stress, GRP78/BiP, an ER protein translocates to the PC3 cell surface and activates NRF2 by a noncanonical NRF2 activation pathway (Bellezza et al., 2017). Sequential treatment of vitamin C and quercetin (Abbasi et al., 2021), or puerarin (J. Li et al., 2021) reduced NRF2 signaling in advanced staged PCa cells. In the TRAMPC mouse model and in the TRAMPC1 PCa cells, expression of NRF2 is suppressed due to promoter methylation (S. Yu et al., 2010), which can be reversed by sulforaphane (Ferreira et al., 2018; C. Zhang et al., 2013), a synthetic curcumin analogue (Khor et al., 2011; W. Li et al., 2016), 3,3′-diindolylmethane (T. Y. Wu et al., 2013), and Indole-3-carbinol (T. Y. Wu et al., 2012) to enhance NRF2 expression and reduce PCa tumor size in TRAMPC PCa mice. The antibiotic, salinomycin, increases apoptosis via ER and oxidative stress in advanced PCa by suppressing NRF2 activity and expression of its target genes: HO-1, NQO1 and GCLC (J. Yu et al., 2021). In TRAMPC mouse models it was observed that the γ-tocopherol-rich diet reduced PCa growth by increasing NRF2 protein expression by reducing the promoter methylation (Huang et al., 2012).

However, many of the pre-clinical studies with NRF2 activators and inhibitors were not mechanistically supported by NRF2 gene overexpression and/or suppression experiments. The chemical or biochemical compounds used for preclinical studies could be nonspecific and may act via other independent NRF2 mechanisms. For example, a commonly used NRF2 activator, sulforaphane, alters growth and metabolic activity of PCa cells and the function of cancer stem like cells (Labsch et al., 2014; Singh et al., 2019; Rutz et al., 2020). However, role of NRF2 were not confirmed in these reports. Sulforaphane may also act via NRF2 independent pathways. Sulforaphane alters AKT/mTOR pathways, inhibits mitochondrial fission by mitigating recruitment of DRP1 on mitochondrial membrane, inhibits inflammasome in NRF2 independent ways (Greaney et al., 2016; O'Mealey et al., 2017; Shawky and Segar, 2017; Y. Zhang et al., 2021). Therefore, specific NRF2 mediated effects of the candidate compounds need to be confirmed by overexpression and/or RNA inhibition mediated methods before concluding the mechanistic properties of the compound are due to only NRF2 mediated in the preclinical studies.

Therefore, from the overall summary (Table 1) of this section, we may surmise that downregulation of NRF2 in aggressive PCa (PC3 and DU145) and upregulation of NRF2 in normal cells surrounding the tumor and early stage PCa cells (LNCaP cells, TRAMPC1 cells and TRAMPC mouse models) may be advantageous against PCa growth. These studies suggest that there are several chemical and organic NRF2 modulators that can successfully modulate NRF2 levels, overall ROS levels and increase the conventional treatment efficacy in PCa. However, proper stage specific administration of activators or inhibitors of NRF2 are needed to ensure the treatment efficacy and reduce side effects and tumor growth in PCa.

**TABLE 1:** Effects of NRF2 modulators used in pre-clinical research in PCa growth regulation.

*In vitro* or *In vivo* PCa models	Up/down regulation of NRF2	Effects of NRF2 modulators	References
DU145 and PC3	Downregulation of NRF2 (by salinomycin)	Decreases cell viability by increased ROS, oxidation of nucleic acids and lipids. Decreases the activity of antioxidative enzymes and increases the unfolded protein response and endoplasmic reticulum stress.	Liang et al. (2019)
LNCaP, PC3 and DU145 cells but not in normal prostate cells	Downregulation of NRF2 (by a polyphenol-rich fraction of *Bergenia ligulate* plant)	Upregulates ROS levels and increases monoamine oxidase A activity and cell death.	(J. Yu et al. (2021))
PC3-tumor xenograft in NOD-SCID	Downregulation of NRF2 (by a polyphenol-rich fraction of *Bergenia ligulate* plant)	Decreased tumor growth alone and in synergy with paclitaxel.	(J. Yu et al. (2021))
Normal testicular cells but not in PC3 cells	Upregulation of NRF2 (by roflumilast)	Decreases the toxicity of cisplatin based PCa therapy by decreasing ROS and inflammation.	(Ghosh et al. (2021))
Normal prostate fibroblasts but not in the PCa cells	Upregulation of NRF2 (by manganese porphyrins)	Decreases ROS, oxidative damage, and fibrosis in normal prostate fibroblasts after radiation and enhances radiation effects on PCa growth reduction.	(Tong et al. (2016); Shrishrimal et al. (2017); Chatterjee et al. (2020))
PCa cells	Downregulation of NRF2 (by inhibition of TUG1, a long noncoding RNA, which enhances NRF2 activity)	Decreased proliferation, migration, and invasion of PCa cells.	Abdel-Wahab et al. (2021)
PC3 cells	Upregulation of NRF2 (by tunicamycin)	In the presence of endoplasmic reticulum (ER) stress, GRP78/BiP, an ER protein that translocates to the cell surface and activates NRF2 and promote PC3 survival.	(J. Y. Chen et al. (2019))
DU145 and PC3	Downregulation of NRF2 (by sequential treatment of vitamin C and quercetin)	Reduces GPx, GR and NQO1 enzymatic activity, increases ROS and suppresses, PCa cell growth.	(G. Yang et al. (2020))
DU145, PC-3, and LNCaP	Downregulation of NRF2 (by puerarin)	Growth inhibitory effect via apoptosis against DU145 and PC-3 cells, whereas slight effect on LNCaP cells. Increases Keap1 expression, declines NRF2, HO-1 and NQO1 expression in DU145 and PC3 cells.	Abbasi et al. (2021)
TRAMPC mouse model and in the TRAMPC1 PCa cells	Upregulation of NRF2 (by sulforaphane, a synthetic curcumin analogue, 3,3′-diindolylmethane, and Indole-3-carbinol	Promoter methylation suppresses NRF2 expression in less aggressive TRAMPC mice and TRAMPC1 cells. Reversal of NRF2 promoter methylation increases tumor cell death and reduction of tumor size.	(Ferreira et al. (2018); Khor et al. (2011); J. Li et al. (2021); W. Li et al. (2016); T. Y. Wu et al. (2013); S. Yu et al. (2010); C. Zhang et al. (2013))
DU145 and PC-3 cells	Downregulation of NRF2 (by salinomycin)	Increases apoptosis via ER and oxidative stress by suppressing NRF2 expression and expression of its target genes.	Liang et al. (2019)
TRAMPC mouse models	Upregulation of NRF2 (by γ-tocopherol)	decreases promoter methylation of NRF2 and prevents PCa growth.	(T. Y. Wu et al. (2012))

## 7 NRF2 modulators in anticancer clinical trials

According to the ClinicalTrials.gov (https://clinicaltrials.gov/), NRF2 modulators are being used in several active and terminated clinical trials for different cancers, including prostate cancer. We have listed the candidates in Table 2. In many of the trials, NRF2 inducers (such as, Sulforaphane) were used to reduce treatment side effects as normal tissue protector, not directly used as an anticancer agent. Most of the examined NRF2 modulators used in the clinical trials are NRF2 inducers. Some of the NRF2 inhibitors, such as, Luteolin, Trigonelline and β-Lapachone are also in trials. There have been no significant anticancer effects reported in these clinical trials yet. Many of the trials used dietary antioxidants as NRF2 activators, such as curcumin. One of the major clinical challenges for dietary antioxidants is bioavailability in the target tissue. The clinical dose of the dietary antioxidant compound may not be achievable in target tissue to induce NRF2.

**TABLE 2:** NRF2 modulators used for clinical trials in different cancers.

NRF2 inducer/inhibitor	Description	Conditions for clinical trial
Sulforaphane (NRF2 inducer)	Sulforaphane in chemoprevention	Bladder cancer
	Nutritional supplementation of sulforaphane on anthracycline related cardiotoxicity in breast cancer	Breast cancer
	Sulforaphane in broccoli sprout extract	
	Topical application of sulforaphane on radiation dermatitis	
	SFX-01 in the treatment and evaluation of metastatic breast cancer	
	Cruciferous vegetable intake in histone status	Colon cancer
	Broccoli sprout extract in recurrence in head and neck squamous cell cancer	Head and neck cancer
	Sulforaphane in lung cancer chemoprevention	Lung cancer
	Pilot study evaluating broccoli sprouts in advanced pancreatic cancer	Pancreatic ductal adenocarcinoma
	Utilizing MRI to study the effect of sulforaphane on prostate cancer	Prostate cancer
	Effect of Sulforaphane on prostate cancer prevention	
	Biomarkers of sulforaphane/broccoli sprout extract in prostate cancer	
	Effects of sulforaphane on normal prostate tissue	
	Chemoprevention of prostate cancer, HDAC inhibition and DNA methylation	
	Sulforaphane for treating recurrent prostate cancer	
Curcumin (NRF2 inducer)	Dietary supplementation of curcumin	Breast cancer
	Curcumin versus placebo combination in first-line treatment of metastatic castration resistant prostate cancer	Metastatic castration resistant prostate cancer
Resveratrol (NRF2 inducer)	Resveratrol in colon cancer	Colon cancer
	Grape-derived low dose resveratrol	
	Resveratrol in early-stage colorectal cancer	
	SRT501 in colorectal cancer hepatic metastases	
	Resveratrol in human hepatocyte function in cancer	Liver cancer
	Resveratrol’s effects on Notch-1 signaling in low grade gastrointestinal tumors	Neuroendocrine tumor
	Effect of resveratrol on serum IGF2 among African American women	Chemoprevention
	Dietary polyphenols and methylxanthines in mammary tissues	Breast cancer
	SRT501 alone or in combination with Bortezomib	Multiple myeloma
	Resveratrol and sirolimus in lymphangioleiomyomatosis trial	Lymphangioleiomyomatosis
	Dietary intervention in follicular lymphoma	Follicular lymphoma
	With pazopanib and paclitaxel in stage III and stage IV melanoma	Stage III melanoma
CDDO (NRF2 inducer)	CDDO to treat lymphomas	Lymphoma
	CDDO treatment with gemcitabine	Pancreatic cancer
Oltipraz (NRF2 inducer)	Oltipraz in the prevention of lung cancer	Lung cancer
Epigallocatechin Gallate (EGCG), (NRF2 inducer)	Chemo preventive effects of EGCG	Colon cancer
	Green tea extracts for the prevention of colorectal cancer	
	Polyphenon E in high-risk of colorectal cancer	
	Effect of green tea extract in metachronous adenomas	
	Green tea catechin extract in localized prostate cancer	Prostate cancer
	Green tea extract in progression of prostate cancer	
	Green tea catechins in men on active surveillance	
	Lycopene or green tea for men at risk of prostate cancer	
	Polyphenon E in men with high-grade prostatic intraepithelial neoplasia	
	Fish oil and green tea extract in preventing prostate cancer	
	EGCG modulate the cytotoxic effects of chemotherapeutic agents in human urothelial carcinoma cells	Breast cancer
	Epigallocatechin-3-gallate (EGCG) for skin prevention in breast cancer receiving adjuvant radiotherapy	
	Green tea catechin extract in treating hormone receptor negative stage I-III breast cancer	
	Green tea and reduction of breast cancer risk	
	Epigallocatechin-3-gallate (EGCG) for esophagus protection in lung cancer receiving radiotherapy	Lung cancer
	Green tea extract in preventing cancer in heavy smokers	
	Oral green tea extract for small cell lung cancer	
	Epigallocatechin-3-gallate (EGCG) in esophageal cancer	
	Green tea extract in nonmetastatic bladder cancer	Bladder cancer
	Green tea extract in preventing cervical cancer in low-grade cervical intraepithelial neoplasia	Cervical cancer
	Green tea extract in multiple myeloma	Multiple myeloma
	Standardized dietary supplement with gemcitabine hydrochloride, paclitaxel, metformin hydrochloride	Pancreatic adenocarcinoma
	Topical green tea ointment in superficial skin cancer	Carcinoma, basal cell
Dimethyl fumarate (DMF) (NRF2 inducer)	Dimethyl fumarate (DMF) in relapsed/refractory chronic lymphocytic leukemia	Chronic lymphocytic leukemia
	Dimethyl fumarate, temozolomide, and radiation therapy in glioblastoma multiforme	Glioblastoma
	Dimethyl fumarate (DMF) in cutaneous T cell lymphoma (CTCL)	Cutaneous T cell lymphoma
Apigenin (NRF2 inducer)	Dietary bioflavonoid supplementation for the prevention of neoplasia recurrence	Colorectal cancer
Luteolin (NRF2 inhibitor)	Effect of luteolin in tongue squamous cell carcinoma cell line	Tongue neoplasms
Trigonelline (NRF2 inhibitor)	Radiation-induced damage	Bone metastasis
Bardoxolone methyl (NRF2 inducer)	Advanced lymphoid malignancies	Lymphoid malignancies
	With gemcitabine in unresectable pancreatic cancer	Pancreatic cancer
β-Lapachone (Tissue specific NRF2 pathway inhibitor)	Effect of ARQ 501 in advanced solid tumors	Advanced solid tumors
	ARQ 501 in subjects with cancer	Cancer
	ARQ 501 in combination with gemcitabine in subjects with pancreatic cancer	Pancreatic cancer
	ARQ-761 treatment in pancreatic cancer in gemcitabine/Nab-paclitaxel chemotherapy	Pancreatic cancer
	Safety and Efficacy Study of ARQ 501	Leiomyosarcoma
	ARQ 501 in combination with docetaxel	Carcinoma
	ARQ 501 in squamous cell carcinoma of the head and neck	Head and neck cancer
	ARQ761 with PARP Inhibitor in refractory solid tumors	Lymphoma
Manganese porphyrin (NRF2 inducer in normal tissue)	Trial of BMX-001 or placebo in head and neck cancer patients	Head and neck cancer
	Safety study of BMX-001 (radioprotector) in anal cancer	Anal cancer
	Trial of newly diagnosed high grade glioma treated with concurrent radiation therapy, temozolomide and BMX-001	High grade glioma

There have been completed clinical trials using NRF2 inducers, such as, sulforaphane, curcumin and epigallocatechin gallate (EGCG) on prostate cancer. No data about prostate cancer reduction is available from those trials. However, there are some data published on the effect of dietary sulforaphane (broccoli sprout extract) and epigallocatechin gallate (green tea extract) on prostate cancer patients. No significant correlation of broccoli sprout extract was found with histone deacetylase activity or prostate specific markers (Z. Zhang et al., 2020). Long term daily consumption of decaffeinated EGCG showed accumulation in the plasma and was well tolerated with no adverse effects or change in body mass index in high-grade PIN, atypical small acinar proliferation in the prostate and in high-risk obese prostate cancer patients without reducing prostate cancer (N. B. Kumar et al., 2017; N. B. Kumar et al., 2015; N. B. Kumar et al., 2016). It is also reported that there were no significant changes in the fatty acid synthase and proliferative marker, Ki-67 after short term EGCG supplementation in the human prostate tissue (Z. Zhang et al., 2016). Other clinical studies did not specifically mention the outcome on prostate cancer. According to our above discussion, the use of NRF2 inducers in the advanced stage of prostate cancer will likely not be beneficial. Therefore, clinical stages of the cancers need to be carefully selected when designing the trials.

Another crucial challenge of using NRF2 inducers or inhibitors are the tissue specific effect to induce NRF2. The canonical use of NRF2 modulators will increase NRF2 both in normal and cancer tissue, which will be beneficial for cancer tissue to grow. To overcome this clinical challenge, tissue specific inducers or inhibitors of NRF2 should be used clinically. Manganese porphyrins and β-Lapachone may be advantageous in this issue. Manganese porphyrin enhances NRF2 in the normal tissue only (Shrishrimal et al., 2017; Chatterjee et al., 2020), which helps to sustain the natural anticancer activity of normal cells and increase anticancer treatment efficacy. β-Lapachone senses the NQO1 levels in the tissue and inhibits NRF2 signaling to maintain homeostasis (Gong Q et al., 2021; Wu et al., 2021; Zhao et al., 2021). As the advanced PCa tissue harbors enhanced NRF2-NQO1 levels as compared to the normal prostate tissue, to cope with increased oxidative stress, β-Lapachone may have a better efficacy to reduce NRF2 selectively in cancer tissue. Therefore, we propose that use of tissue specific NRF2 inhibitors in the advanced PCa will achieve better efficacy of the inhibitor to improve conventional anticancer therapy efficacy. The NRF2 inhibitor alone also has anticancer properties via modulating the mitochondrial metabolism.

## 8 Gap in the knowledge

In PCa, mitochondrial metabolic shift plays a key role in PCa progression and therapy resistance. There have not been enough studies conducted to identify the role of NRF2 in each mitochondria-regulated step during PCa progression. Early and late stage PCa are different in terms of metabolism, oxidative environment and NRF2 activity. The role of NRF2 in the regulation of mitochondrial TCA and OXPHOS enzyme activity needs to be studied extensively in the different stages of PCa. The potential function of NRF2 activators and inhibitors with zinc accumulation and mitochondrial citrate release to inhibit oncogenic transformation of PCa, needs to be further studied. The role of NRF2 in mitochondrial dynamic changes, such as, fission, fusion, mitophagy and biogenesis of mitochondria in different stages of PCa also needs to be studied in PCa cells and in different cell types in the PCa tumor microenvironment.

## 9 Conclusion

**FIGURE 3:**
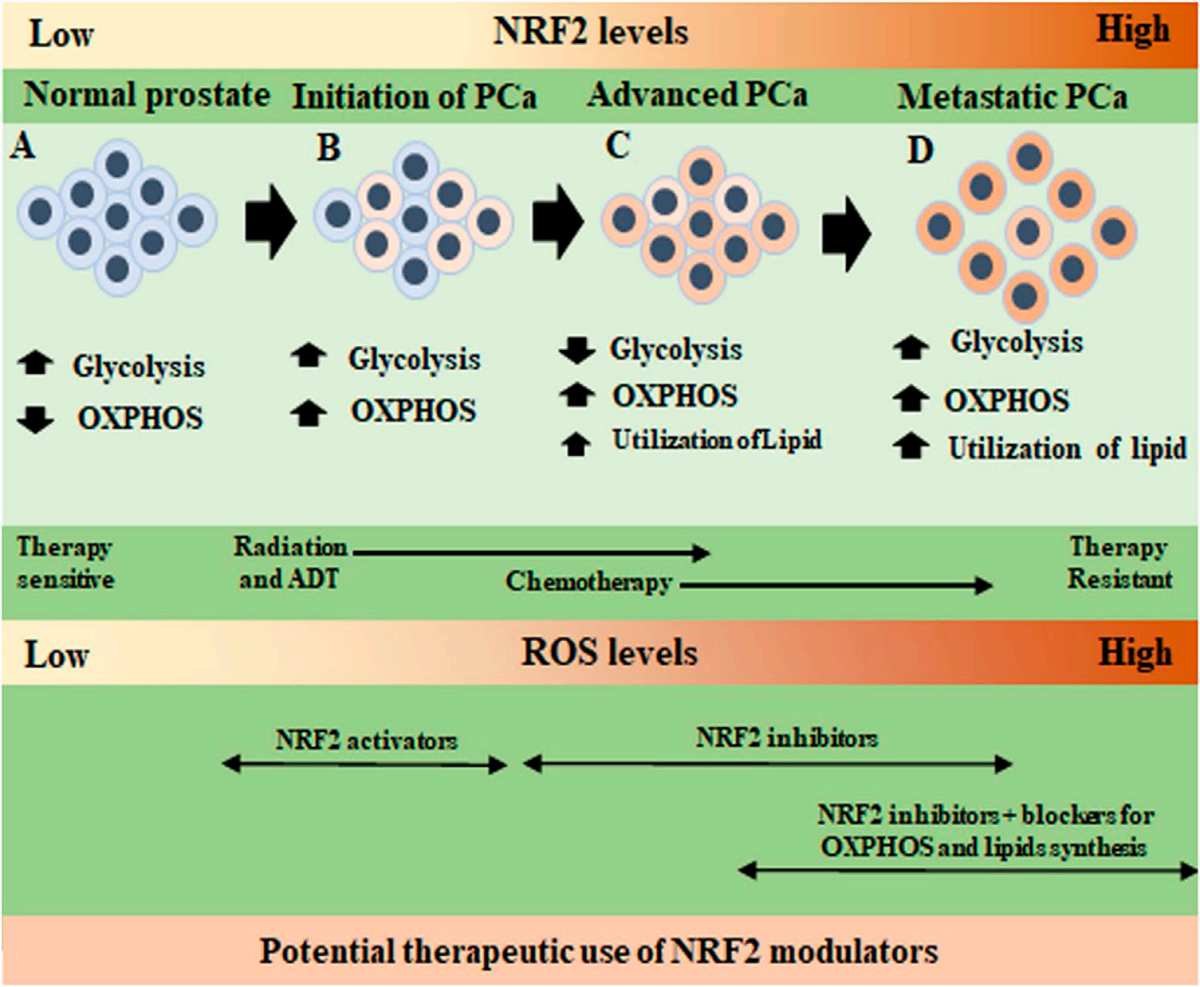
Specific regulatory action of NRF2 on metabolic changes in the different stages of prostate cancer progression. **(A)**. In normal prostate epithelium, NRF2 and ROS levels are low, and cells use glycolysis for energy production with a low basal level of oxidative phosphorylation (OXPHOS). **(B)**. In the initiation of prostate cancer (PCa), cells slowly shift to OXPHOS to produce most of the required energy. Glycolysis remains in a lower level. At this stage, radiotherapy (RT), and androgen deprivation therapy (ADT) begin. Both RT and ADT increase ROS to kill the cancer cells. Therefore, before RT and ADT, the use of a NRF2 activator may reduce basal ROS levels of the cells and push them to non-transformed mode of metabolism. After initial cell death due to RT and ADT, surviving PCa cells adapt to a high ROS environment and NRF2 levels increase in the PCa cells. **(C)**. In advanced stages PCa, cells fully use OXPHOS, reduce glycolysis and after chemotherapy they begin using lipids as another energy source (smaller font size as cells do not depend on lipid only). Overactivation of OXPHOS produces more ROS and NRF2 levels increases. The use of NRF2 inhibitors as a therapeutic molecule will inhibit PCa cell growth in a high ROS environment. **(D)**. In the metastatic therapy resistant PCa, cells reactivate glycolysis by noncanonical reactivation of AR signaling. In this high energy demanding stage, PCa cells use glycolysis, OXPHOS, and lipid metabolism to survive (bigger font size as lipid utilization increased). Cells are dependent on NRF2 to combat ROS. In this stage, use of NRF2 inhibitors along with conventional OXPHOS and lipid pathway inhibitors with chemotherapeutic agents may be useful to control therapy resistant PCa growth and increase therapy efficacy.

NRF2 activity cannot be specifically defined as cytoprotective or pro-tumorigenic in a PCa tumor environment. Many factors including the localization of NRF2 in the tumor tissue, the stages of cancer, the therapy response of the cancer, and the metabolic properties of the cancer determine the anti-tumor or pro-tumor activity of NRF2. In Figure 3, we summarize that NRF2 activators at the early stages of PCa may be a beneficial therapeutic option for PCa. However, in the advanced stages of PCa, NRF2 inhibitors, along with the conventional PCa-stage specific therapies, may be more advantageous for PCa regulation. Without considering these issues, the blanket use of NRF2 activators or inhibitors in PCa treatment could be deleterious for PCa patients.

## Abbreviations

ADT, androgen deprivation therapy; AR, Androgen receptor; ARE, antioxidant responsive element; ATP, adenosine triphosphate; BPH, benign prostatic hyperplasia; CRPC, castration resistant prostate cancer; CSC, cancer stem cell; DHT, dihydrotestosterone; DNA, deoxyribonucleic acid; KEAP1, kelch-like ECH-associated protein 1; MnSOD, manganese superoxide dismutase; MSC, mesenchymal stem cell; NEPC, neuroendocrine prostate cancer; NOD-SCID mice, nonobese diabetic/severe combined immunodeficiency; NRF2, nuclear factor erythroid 2-related factor 2; OXPHOS, oxidative phosphorylation; PCa, prostate cancer; PCPT, prostate cancer prevention trial; PIN, prostatic intraepithelial neoplasia; ROS, reactive oxygen species; TCA cycle, tricarboxylic acid cycle.
